# Why and when does multitasking impair flow and subjective performance? A daily diary study on the role of task appraisals and work engagement

**DOI:** 10.3389/fpsyg.2024.1384453

**Published:** 2024-07-25

**Authors:** Helen Pluut, Maral Darouei, Marijn Eveline Lidewij Zeijen

**Affiliations:** ^1^Department of Business Studies, Institute of Tax Law and Economics, Leiden University, Leiden, Netherlands; ^2^Department of Management and Organization, School of Business and Economics, VU Amsterdam, Amsterdam, Netherlands; ^3^Department of Social, Health and Organizational Psychology, Utrecht University, Utrecht, Netherlands

**Keywords:** multitasking, flow, engagement, stress appraisal, experience-sampling methodology

## Abstract

In this diary study, we contribute to research on day-level multitasking in organizations by investigating why and when multitasking impairs employees’ work-related flow and subjective job performance on a daily basis. Drawing on Lazarus and Folkman’s transactional model of stress and coping, we propose that employees’ appraisal of their daily tasks (i.e., less challenging and more hindering) may explain why multitasking has negative implications for flow and job performance. Moreover, we expect that daily work engagement can buffer the detrimental effects of multitasking on flow and job performance. A total of 33 professional workers in the food industry participated in our study and were asked to respond to 10 daily surveys at work across 4 weeks. In line with our expectations, results showed that on days when employees’ working time was highly fragmented across a high number of tasks, they experienced less flow and, in turn, their job performance was lower on that particular day. Moreover, appraisal of daily tasks as less challenging – though not more hindering – explained why multitasking impairs flow. Finally, daily work engagement buffered the detrimental impact of multitasking on flow. The results presented in this paper offer novel and ecologically valid insights into why and when multitasking may backfire for employees.

## 1 Introduction

People believe that doing several things at the same time will help them get more done, and by switching among tasks, they feel productive as more tasks are performed in a single day (Adler and Benbunan-Fich, 2013; Peifer and Zipp, 2019). Yet this might simply be an illusion, also referred to as the myth of multitasking (Rosen, 2008) or multitasking paradox (Appelbaum et al., 2008). Concerns about productivity loss due to multitasking are rampant, especially with the alarming new heights that it has reached. In a field study by Mark et al. (2005), almost a quarter of interrupted work was not resumed on the same day. If work was resumed, it took more than 25 min and employees had worked in more than two other working spheres before resuming interrupted work. Wajcman and Rose’s (2011) study documented an average of 88 work episodes per day, with not even half of the workday being spent on activities that last for more than 10 min. The COVID-19 pandemic posed additional challenges, as employees working from home reported an increase in multitasking behaviors (Leroy et al., 2021). As Leroy et al. (2021) put it: “work time has never been so fragmented” (p. 1457).

Scholars are yet to fully understand the implications of multitasking for employees and organizations. If detailed comprehension is lacking, society runs the risk that multitasking remains common organizational practice without proper management of its complexities. To increase organizations’ willingness and ability to address these concerns, it is imperative to study how multitasking behavior relates to employee effectiveness. After having been dominated by studies conducted in laboratory settings (see Baethge et al., 2015), the field has witnessed a rise in diary studies, both quantitative (e.g., Aitken et al., 2023) and qualitative (e.g., Feldman and Greenway, 2021). Importantly, a few diary studies suggest that daily flow – a short-term peak experience that individuals experience when fully immersed in an activity (Csikszentmihalyi, 1997) – may be a key mechanism in the day-level multitasking–job performance relationship (Peifer and Zipp, 2019; Aitken et al., 2023). Yet it remains elusive what are the psychological processes underlying employees’ responses to multitasking.

Against this background, we develop and test a conceptual model that specifies a pathway (i.e., *why*) and condition under which (i.e., *when*) day-level multitasking behavior impairs work-related flow and ultimately hampers the subjective performance of employees. In building our conceptual model, we draw on the transactional model of stress and coping (Lazarus and Folkman, 1984). This theoretical model posits that reactions to work stressors, such as fragmentation of work time due to multitasking, depend on (1) how we appraise those stressors (i.e., as opportunity or threat) and (2) the resources we possess to cope with those stressors. Recent research on interruptions has indeed shown that the (positive or negative) appraisals of employees shape reactions (Hunter et al., 2019; Darouei et al., 2024). We go one step further and argue that a work stressor can also shape the appraisal of other, more neutral job characteristics. Specifically, we propose that multitasking influences employees’ appraisal of their daily tasks (i.e., as less challenging and more hindering) and this explains why multitasking behaviors would impair flow. Moreover, we explore whether day-specific work engagement – a positive and high arousal affective-motivational state characterized by energy and involvement (Bakker et al., 2011) – as a resource can help employees cope with multitasking.

Our study contributes to the literature on multitasking, stress, and flow in at least three notable ways. First, we put forward flow as a key mediator that can explain the relationship between multitasking and job performance. The multitasking reality of modern organizations suggests that interruptions to workflow are an accepted part of organizational life (Jett and George, 2003). Yet, surprisingly, the concept of flow has received hardly any attention in research on multitasking and work interruptions (see the review by Puranik et al., 2020). Our study is among the first to examine the day-to-day relationship between multitasking and flow [for related work, see Peifer and Zipp’s (2019) study on multitasking behaviors, and Aitken et al.’s (2023) on intrusions while teleworking]. Second, we propose that appraisal is key to understanding the psychological processes that may explain how multitasking influences employees’ well-being and job performance. We link daily fluctuations in multitasking behaviors to meaningful variations in task appraisals in order to illuminate the stressor–well-being relationship (see also Smith et al., 2022). Our third contribution relates to the examination of work engagement as a day-level variable that explains why individuals may experience multitasking differently across days. No prior studies have yet investigated whether and how work engagement can assist employees in handling daily job demands, even though it is a source of energy and concerns the investment of personal resources (Christian et al., 2011). While work engagement is typically studied as an outcome in and of itself (Puranik et al., 2020) or as a mediator in the association between job characteristics and performance (Bakker et al., 2023), we examine work engagement as a day-specific resource that can buffer the detrimental effects of multitasking behaviors on flow and job performance.

## 2 Theoretical framework

We draw on the two-dimensional typology of multitasking behaviors to conceptualize multitasking along the lines of *share of resources* allocated to the execution of work activities and *share of time* in which work activities are observed (Circella et al., 2012). With regard to the time dimension, our focus is on multitasking within the time frame of a single working day; that is, all tasks performed during the day will be considered to have happened sequentially or simultaneously (see Kirchberg et al., 2015). Monotasking refers to the situation in which a single activity occupies a person’s full resources for a particular period of time. Within the time frame of a full working day, monotasking is very uncommon nowadays, since many – if not most – employees are forced to shift attention between a high number of tasks on a daily basis. When employees execute multiple tasks, their behavior can be classified as switching, interleaving, or overlaying, depending on a person’s allocation of (e.g., mental) resources among the tasks of a particular workday (Circella et al., 2012). Switching is alternating between activities in such a way that one fully interrupts one task with another. Interleaving involves partial alternation to a second task, while another activity remains in the background. Hence, in the case of interleaving, the main activity claims most but not all resources, for instance due to attention residue (Leroy, 2009). Overlaying refers to the simultaneous execution of tasks: both activities are carried out at the same time with a parallel allocation of resources. Whether it happens sequentially or simultaneously, multitasking can lead to fragmentation of the work day (Appelbaum et al., 2008), and the question central to our study is “at what cost?”

In this diary study, we integrate key theoretical models of stress with the literature on multitasking and flow in order to elucidate the process by which day-level multitasking impedes job performance. Carrying out a task can be seen as goal-directed behavior, and doing more than one thing at a time (be it sequentially or simultaneously) is associated with regulation hindrances that act as stressors (Frese and Zapf, 1994). In the transactional model of stress and coping (TSC), Lazarus and Folkman (1984) viewed stress as an individual outcome generated through the person’s appraisal of stressors in the environment. When we encounter a stressor, we first assess how stressful it is through primary appraisal. We simultaneously engage in secondary appraisal, evaluating whether we have the necessary resources to cope with the stressful situation. Building on the TSC, the challenge-hindrance stressor framework (CHSF; LePine et al., 2005) postulates that stressors are conceptually distinct from each other, such that some stressors tend to be appraised as challenges (i.e., potential for achievement and personal growth), while other stressors tend to be appraised as hindrances (i.e., may thwart personal development). Interestingly, Peifer and Zipp (2019) relied on a model that integrates flow into the transactional model of stress, which they refer to as the transactional model of stress and flow (TMSF). This adapted model explains the connection between stressors and flow. When individuals perceive stressors as challenges (primary appraisal) and possess adequate coping resources (secondary appraisal), they are more likely to experience flow as an alternative state to stress.

The concept of flow finds it origins in the work of Mihaly Csikszentmihalyi. He studied the subjective experiences of creative painters, chess players, rock climbers, and many others, and he was intrigued when these people became fully absorbed in their activity and found that activity intrinsically motivating. People “in flow” are in a state of consciousness where they become totally immersed in an activity and operate at full capacity (Nakamura and Csikszentmihalyi, 2002). People can find flow in almost any activity, but research shows it is mainly experienced in the work environment (Csikszentmihalyi and LeFevre, 1989), where flow refers to peak experiences of fluent, uninterrupted work (Gerpott et al., 2022).

**Figure 1:**
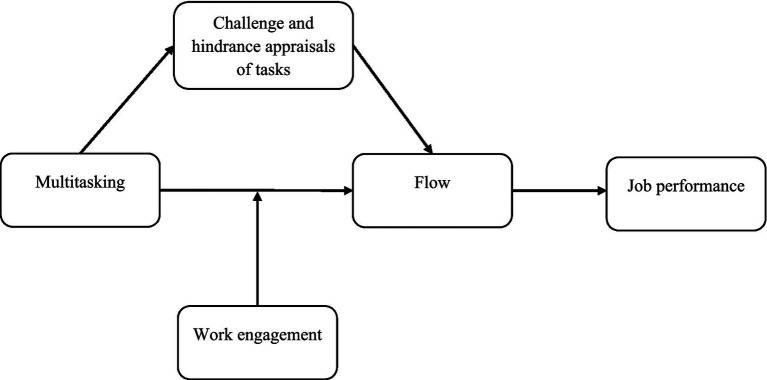
Conceptual model.

We draw on Lazarus and Folkman’s (1984) TSC and its extensions (CHSF and TMSF) to propose that day-level multitasking is a hindrance stressor that negatively impacts the primary appraisal of one’s daily tasks, ultimately influencing flow experiences and daily performance. We also propose that multitasking interferes with the experience of flow less strongly when employees feel engaged at work. Work engagement is a motivational construct that refers to the investment of personal resources toward the tasks associated with one’s work role (Christian et al., 2011). In contrast to flow, which is more closely related to a specific activity as it is an optimal experience of fluent work (i.e., experiential well-being; Ilies et al., 2017), work engagement represents a psychological connection with one’s work in general (Christian et al., 2011; Gerpott et al., 2022) (see also Yan and Donaldson, 2023, on the differences between flow and engagement). While engagement is a relatively enduring state of mind, it also ebbs and flows, showing day-to-day fluctuations around an employee’s average level (Sonnentag et al., 2010). Applying the TMSF, we argue that state work engagement will allow for a more positive secondary appraisal in stressful situations of high multitasking such that on days when employees are engaged, they come to experience flow as an alternative experience to stress. The overall conceptual model guiding this research is depicted in Figure 1.

### 2.1 Hypotheses

Multitasking may be an impediment to flow. Task-related preconditions for the experience of flow are a balance between challenges and skills, clear and specific goals, and immediate feedback on one’s performance (see Fullagar and Kelloway, 2013). Under these conditions, experience naturally unfolds from moment to moment and one enters a flow state (Nakamura and Csikszentmihalyi, 2002). However, the cognitive costs of executing multiple tasks in parallel and alternating between tasks may add considerably to the demands and challenges of a particular workday. Challenge stressors may turn into hindrance stressors when interruptions accumulate (Baethge et al., 2015). Moreover, alternations imply that employees are shifting goals, and any feedback from the work itself is suspended until the moment of resumption and completion of the main task (Monk et al., 2008). Thus, multitasking interferes with the preconditions of flow.

In addition, the very nature of multitasking is contradictory to how the flow experience is characterized. Flow is a subjective state with the following characteristics: (a) intense and focused concentration, (b) loss of self-consciousness, (c) a sense of control, (d) distortion of time, and (e) intrinsic rewards (Nakamura and Csikszentmihalyi, 2002; Fullagar and Kelloway, 2013). When work is fragmented due to multitasking, it becomes difficult to get fully absorbed in one’s work. After all, deep involvement in the task at hand is interrupted and disrupted. Multitasking may also limit one’s sense of control over the processes and outcomes of the task because many alternations are uncontrollable and require the expenditure of self-regulatory resources (Freeman and Muraven, 2010). Ready-to-resume interventions may work (Leroy and Glomb, 2018), however, as Fullagar and Kelloway (2013) noted, “as soon as attention shifts to try to maintain control, flow dissipates” (p. 44). Furthermore, when people multitask, it is very unlikely that the notion of time disappears and that employees will feel time is flying by. Instead, multitaskers are very much aware of the notion of time, as indicated by their higher sense of time pressure (Baethge and Rigotti, 2013; Leroy and Glomb, 2018).

To experience the peak state of flow at work, an initial investment of self-regulatory resources is required (Csikszentmihalyi, 2000). Interestingly, research shows that people sometimes voluntarily switch between tasks when they are unable to achieve the state of flow in an ongoing task (e.g., when they are stuck; Adler and Benbunan-Fich, 2013). Although we acknowledge that a switch can enhance flow, based on the aforementioned, we posit that cumulative switches during the day will make it difficult to enter or stay in a flow state. Thus, we hypothesize that on days when employees experience higher levels of multitasking, they will report reduced flow compared with days when they multitask to a lesser extent (see also Peifer and Zipp, 2019).

*Hypothesis 1*: Within individuals (across days), multitasking is negatively related to flow experienced at work.

We also propose a mediating mechanism for the within-individual relationship between multitasking behaviors and flow, namely appraisal of the daily tasks as challenging or hindering. No single day is the same; that is, employees work on a different set of tasks every day. Importantly, how employees appraise these tasks may vary across days. Although tasks are stressors, “many everyday stressors are not clearly positive nor negative and so are most likely to be open to personal appraisal” (Hobfoll, 1989, p. 519). In line with this notion, scholars have distinguished between challenge and hindrance stressors (LePine et al., 2005).

We believe that primary appraisal of the daily tasks may explain why multitasking behavior has negative implications for flow. Doing more than one thing at a time is not harmful *per se*, but employees may find it difficult to interpret their tasks, on average, as opportunities to grow either personally or professionally when they have to perform multiple tasks in parallel (i.e., overlaying) or alternate between tasks (i.e., switching and interleaving). Any single task that would under normal circumstances be considered a challenge stressor may become a hindrance stressor when an employee has limited opportunity to work on it with undivided attention. Thus, we expect that the higher the level of multitasking on a particular day, the less likely it is that an individual will appraise the sum of daily tasks as a positive challenge and the more likely it is that the daily tasks are jointly appraised as a hindrance.

These appraisals, in turn, should be related to flow. The TMSF posits that flow is experienced during a task that is appraised as challenging (Peifer and Zipp, 2019). Previous between-individual research has found that challenge demands are positively related to flow, while hindrance demands are negatively related to flow (Van Oortmerssen et al., 2020). If employees see the potential for personal growth and gain in executing their tasks, they may come to experience “eustress” (Selye, 1983), an experience of being totally focused in a mindful state of challenge and a healthy state of aroused attention on the task (Hargrove et al., 2013). A difficult-yet-manageable set of tasks will help employees savor their work, find the work experience of that day rewarding, and stay immersed in the tasks at hand. In contrast, looking upon one’s work as stressful may seriously undermine a flow state. Thus, we hypothesize that task appraisal mediates the relationship between multitasking and flow in such a way that multitasking is negatively associated with challenge appraisal, which in turn is positively associated with flow (H2a), while multitasking is positively associated with hindrance appraisal, which in turn is negatively associated with flow (H2b).

*Hypothesis 2a*: Challenge appraisals of the daily tasks will mediate the within-individual effect of multitasking on flow.

*Hypothesis 2b*: Hindrance appraisals of the daily tasks will mediate the within-individual effect of multitasking on flow.

Much research has been devoted to understanding when and for whom multitasking inhibits well-being and performance (Puranik et al., 2020). Day-specific moderators, however, seem a neglected focus. We argue that people’s ability to deal with multitasking can be higher on some days than on other days, dependent on one’s daily level of work engagement. On days when employees are engaged, they feel energetic and are in a positive, fulfilling work-related state of mind (Schaufeli et al., 2002). When they are in this state, they are better able to channel physical, emotional, and cognitive energies into their work tasks such that they are not easily fatigued and can show persistence in the face of difficulties (Christian et al., 2011). A workday full of multitasking is cognitively and emotionally demanding and likely to deplete self-regulatory resources, with little opportunity for recovery and replenishment (Leroy, 2009; Freeman and Muraven, 2010; Baethge and Rigotti, 2013; Baethge et al., 2015). The state of work engagement offers substitute personal resources that should make it easier to deal with the stressful nature of multitasking. Applying insights from the TMSF (Peifer and Zipp, 2019) to our model, we posit that work engagement offers the coping resources necessary to experience flow as an alternative experience to stress. Thus, we expect that on days when employees feel energetic and dedicated at work, they are rather well-equipped to retain a high level of concentration and absorption in the face of multitasking.

*Hypothesis 3*: Work engagement moderates the within-individual effect of multitasking on flow such that on days when employees are highly engaged, the negative effect of multitasking on flow is weaker compared with days when employees are less engaged.

When employees become totally immersed in their work and enjoy it intensely, they are more likely to excel at what they do. During flow, performance is automatic, and one has a sense of confidence and ease (Harris et al., 2017). Flow is a peak experience that often coincides with optimal performance (Csikszentmihalyi, 1997). Bakker and Van Woerkom (2017) argued that flow is a desirable state not only for task performance but also for creativity, productivity, and service quality. In a cross-sectional field study, Demerouti (2006) showed that work-related flow was beneficial for both in-role and extra-role performance, as rated by colleagues, but only for conscientious employees, who apparently are better at directing their attention toward achieving crucial tasks that are in line with the goals of the organization. The flow–performance relationship has been established also at the within-individual level, with a number of daily diary studies linking the flow state to both in-role and extra-role performance (Peifer and Zipp, 2019; Soriano et al., 2021; Weintraub et al., 2021; Gerpott et al., 2022; Aitken et al., 2023). These findings have been further corroborated in a recent meta-analysis by Liu et al. (2023), who demonstrated that flow is positively related to job performance.

Based on the logic above, we hypothesize that on days when employees experience high levels of flow, they will feel they are performing better compared with days when they are not in a state of flow. As indicated in previously formulated hypotheses, we expect that multitasking will negatively influence flow through the mediating mechanism of task appraisal. Therefore, we also hypothesize a serial mediation such that multitasking has an indirect effect on job performance via challenge and hindrance appraisals of the daily set of tasks and subsequently flow.

*Hypothesis 4*: Within individuals (across days), flow is positively related to job performance.

*Hypothesis 5*: Challenge and hindrance appraisals of the daily tasks and flow will serially mediate the within-individual relationship between multitasking and job performance.

## 3 Materials and methods

### 3.1 Sample and procedure

To empirically test the proposed model, we designed a diary study of work activities aimed to capture fragmentation of work time due to multitasking as it appears in everyday organizational life. The data for this study were collected at a multinational company that is world leading in the food industry. We collected data among employees of a business unit situated in the Netherlands. To get a representative view of employees’ multitasking, flow experience, work engagement, appraisal of daily tasks, and performance, daily measurements were repeated for a total of 10 days spread evenly across 4 weeks of data collection. Daily surveys were sent out to 65 employees who agreed to participate in this study. A response rate of 75.4% resulted in 49 employees completing the daily surveys, capturing a total of 189 daily records. Participants were instructed to complete the survey toward the end of their workday. Records that were not completed at the designated time were removed for further analyses. Moreover, as we aimed to study daily fluctuations in multitasking and other constructs, we also had to remove respondents with only a single daily record. Our final sample consisted of 158 daily records from 33 employees.

Only 27 of the 33 employees completed a one-time questionnaire at the end of the study, which contained questions on demographic variables. We have missing data for three of these participants, thus resulting in descriptive information for 24 respondents, which consisted of 11 women (45.8%) and 13 men (54.2%), with a mean age of 35.58 years (*SD* = 7.73). Analysis of this descriptive information revealed that, on average, participants had worked 4.96 years within this organization (*SD* = 2.16). Our respondents can be characterized as highly educated, as all had finished higher education, with 91.7% having a master’s degree. The majority of respondents (91.7%) had a fixed contract. The sample included respondents from a variety of different countries, with the majority being French (58.3%) and 16.7% being Dutch. Other nationalities were Afghan, British, Finnish, Greek, Mexican, Polish, and Russian.

### 3.2 Measures

#### 3.2.1 Multitasking

Respondents were asked to provide a list of all their work activities that day and to indicate for each of the activities how much time they spent working on it. In line with the two-dimensional conceptualization of multitasking (Circella et al., 2012), this strategy enabled us to operationalize day-level multitasking as the share of resources (here: time) allocated to work activities within the time frame of a single working day. Focusing on the workday as the unit of time is too coarse to assess the types of multitasking behavior (i.e., switching, interleaving, or overlaying), but our goal here is to get an indication of how fragmented the workday has become due to the execution of multiple and different activities, irrespective of whether tasks are executed in parallel or whether a task is fully or only partly left behind when alternating. We therefore used a diversity measure that captures fragmentation of time across multiple tasks, which was computed using Simpson’s (1949) diversity formula:

$$1-D=\frac{\sum_{i=1}^{R}n_{i}\left(n_{i}-1\right)}{N\left(N-1\right)}$$

Here, *i* represents a particular task, *R* is the total number of tasks, *n*_i_ is the proportion of time spent on the *i*th task, and *N* is the total amount of time spent across all tasks. In this sample, 15 was the highest number of tasks on a day, and an average working day consisted of 6.4 tasks. The value of *D* ranges between 0 (all working time is devoted to a single task – that is, monotasking) and 1 (multitasking in a highly fragmented manner). Simpson’s index captures the level of multitasking for an individual respondent on a given day.

#### 3.2.2 Flow

To measure employees’ daily flow experiences, we used the Flow State Scale (FSS) developed by Jackson and Marsh (1996). This scale consists of a total of 36 items on nine dimensions of the flow state. When conducting diary studies, Ohly et al. (2010) recommend using short scales or even single-item measures. We therefore focused on the subscales ‘concentration’ and ‘autotelic experience’ and selected three items based on factor loadings and face validity. Example items are “I had total concentration today” and “I really enjoyed today’s work experience.” We asked respondents to indicate their agreement on a five-point Likert scale ranging from 1 = *strongly disagree* to 5 = *strongly agree*. Across days, the average internal consistency was 0.79.

#### 3.2.3 Job performance

We evaluated employees’ daily performance at work using a single-item self-report measure. Respondents were asked to indicate their agreement with the following statement: “Today, I was able to carry out the core parts of my job.” Answers were recorded on a five-point Likert scale ranging from 1 = *strongly disagree* to 5 = *strongly agree*.

#### 3.2.4 Work engagement

To assess employees’ daily engagement, we relied on Breevaart et al.’s (2012) validated scale for measuring *state* work engagement and selected only those items that are part of the ultra-short version of the Utrecht Work Engagement Scale (UWES-3; Schaufeli et al., 2019). The UWES consists of vigor, dedication, and absorption as dimensions of engagement. We used one item for each dimension, namely “Today, I felt bursting with energy” (vigor), “Today, I was enthusiastic about my job” (dedication), and “Today, I was immersed in my work” (absorption). We asked respondents to indicate their agreement with these statements on a five-point Likert scale ranging from 1 = *strongly disagree* to 5 = *strongly agree*. Given the conceptual overlap between absorption and flow (Schaufeli et al., 2002), particularly on a daily level, we decided to drop the third item for the specific purposes of our study. The two-item measure of daily engagement had an average Cronbach’s alpha of 0.54 across days.

#### 3.2.5 Appraisal of daily tasks

As mentioned earlier, we asked respondents to provide a list of all their work activities on a particular day. Respondents were also asked how they appraised each of their work activities. We built on prior work on stress appraisal by Searle and Auton (2015), who proposed that appraisal scales can be framed in different ways, amongst others in relation to a task the respondent is currently performing. We then used the response scale of the Valencia Eustress-Distress Appraisal Scale (VEDAS) developed by Rodríguez et al. (2013) to measure task appraisals. Specifically, each task had two corresponding six-point Likert scales that enabled respondents to indicate their positive and negative appraisals of their daily tasks. The response scale for challenge appraisal referred to a task as 1 = *very definitely is NOT a source of opportunity/challenge* or 6 = *very definitely IS a source of opportunity/challenge*, while the response scale for hindrance appraisal referred to a task as 1 = *very definitely is NOT a source of pressure* or 6 = *very definitely IS a source of pressure*. To obtain scores on the degree of challenge and hindrance employees perceived in the total of tasks performed during the workday, we aggregated the task-level challenge and hindrance appraisals of a particular day.

### 3.3 Analyses

The use of repeated measurements resulted in a nested data structure, where days (Level 1; *n* = 158) are nested within individuals (Level 2; *n* = 33). For each variable, we estimated a two-level null model (i.e., no predictors) that partitions the total variance into between-individual and within-individual variance components. Table 1 shows that the percentages of variance due to within-individual variation in construct scores varied between 53.0% (hindrance appraisal) and 94.6% (multitasking). Thus, our constructs show high day-to-day fluctuations, and within-individual analyses are thus appropriate. We therefore use hierarchical linear modeling (HLM; Bryk and Raudenbush, 1992).

**Table 1:** Variance components of null models for level-1 variables.

Dependent variable	Within-individual variance (*σ*^2^)	Between-individual variance (*τ*^2^)	Percent variability within individuals
Multitasking	0.021	0.001	94.6
Flow	0.486	0.159	75.3
Work engagement	0.596	0.246	70.8
Challenge appraisal	0.352	0.152	69.8
Hindrance appraisal	0.310	0.275	53.0
Job performance	0.965	0.242	80.0

Percent variability within individuals was computed as *σ*^2^/(*σ*^2^ + *τ*^2^) * 100. All within-individual variances were significantly different from zero (*p* < 0.001).

To avoid an overly piecemeal analysis of our model, we used the multilevel modeling approach outlined by Bauer et al. (2006) to test our mediation hypotheses. This methodology estimates simultaneously the distinct paths in a mediation model. In all HLM analyses, we specified random intercepts – random slopes for the models at level 2 to account for differences in slopes across individuals. We centered each level-1 predictor variable relative to the individuals’ means across days on that variable. As such, the scores represent deviations from the respondent’s respective mean, and “the subject serves as his or her own control” (DeLongis et al., 1988, p. 487).

## 4 Results

Table 2 presents the descriptive statistics for all study variables as well as the between- and within-individual correlations.

**Table 2:** Correlation matrix.

Variable	*M*	*SD*	1	2	3	4	5	6
1. Multitasking	0.74	0.08	–	−0.24***	−0.08	−0.37***	−0.09	−0.07
2. Flow	3.33	0.52	0.004	–	0.59***	0.33***	0.11	0.48***
3. Work engagement	3.24	0.68	0.12	0.71***	–	0.16	0.11	0.23*
4. Challenge appraisal	3.75	0.51	0.12	0.28	0.16	–	0.23^†^	0.17^†^
5. Hindrance appraisal	3.66	0.62	0.26	−0.42	−0.52**	−0.05	–	0.06
6. Job performance	3.47	0.73	−0.02	0.49**	0.26	0.07	0.03	–

Means (M) and standard deviations (SD) are between-individual descriptive statistics. The correlations below the diagonal represent between-individual associations, which are calculated based on individuals’ aggregated scores (*n*s = 32–33, pairwise). The correlations above the diagonal represent within-individual associations and are calculated using the group-mean centered scores (*n*s = 141 to 156, pairwise). ^†^*p* < 0.10, *p < 0.05, **p < 0.01, ***p < 0.001.

To test Hypothesis 1, we regressed flow on multitasking. We found that on days characterized by high levels of multitasking, employees experienced less flow compared with days on which they had to multitask to a lesser extent (*B* = −1.09, *p* < 0.001, *β* = −0.23). We then used the multilevel procedures of Bauer et al. (2006) to holistically test a 1–1–1 mediation model in which multitasking influences flow via appraisal of daily tasks. We observed that multitasking was negatively associated with appraisal of the sum of daily tasks as challenging (*B* = −1.40, *p* < 0.001, *β* = −0.34), and challenge appraisals were positively associated with flow (*B* = 0.28, *p* = 0.017, *β* = 0.24). Thus, both paths of the mediation were significantly different from zero (see also Model 1 in Table 3). Yet, to test our mediation hypothesis directly, we used a package called ‘RMediation’ (Tofighi and MacKinnon, 2011), which provides an estimate of the indirect effect and a confidence interval around this effect on the basis of the distribution-of-the-product method. RMediation estimated the indirect effect at −0.396 with a 95% CI [−0.821, −0.075], which supports Hypothesis 2a; on days when employees multitasked, they were less likely to appraise their daily tasks as challenging, which interfered with their flow experience. To test Hypothesis 2b, we specified an alternative model with hindrance appraisal as the mediator (Model 2 in Table 3). Within individuals, multitasking was unrelated to appraisal of the sum of daily tasks as hindering (*B* = −0.66, *p* = 0.182, *β* = −0.17), and hindrance appraisals were not linked to flow (*B* = 0.11, *p* = 0.353, *β* = 0.09). We can conclude that the association between multitasking and flow was not mediated by appraisal of daily tasks as hindering, and we, therefore, reject Hypothesis 2b.

**Table 3:** HLM results for testing mediation.

	Mediation model 1				Mediation model 2				Mediation model 3			
	Y: Flow				Y: Flow				Y: Job performance			
	X – M1		M1 – Y		X – M2		M2 – Y		X – M3		M3 – Y	
Level-1 predictors	*B*	*SE*	*B*	*SE*	*B*	*SE*	*B*	*SE*	*B*	*SE*	*B*	*SE*
Intercept	3.79**	0.08	3.35**	0.09	3.64**	0.10	3.37**	0.09	3.36**	0.09	3.45**	0.12
Multitasking (X)	−1.40**	0.37			−0.66	0.49			−1.19**	0.33		
Challenge appraisal (M1)			0.28*	0.11								
Hindrance appraisal (M2)							0.11	0.12				
Flow (M3)											0.69**	0.11

B, unstandardized HLM coefficient; SE, standard error. The X – M and M – Y models were estimated simultaneously with Bauer et al.’s (2006) procedures in HLM 7. *p < 0.05, **p < 0.01.

**Figure 2:**
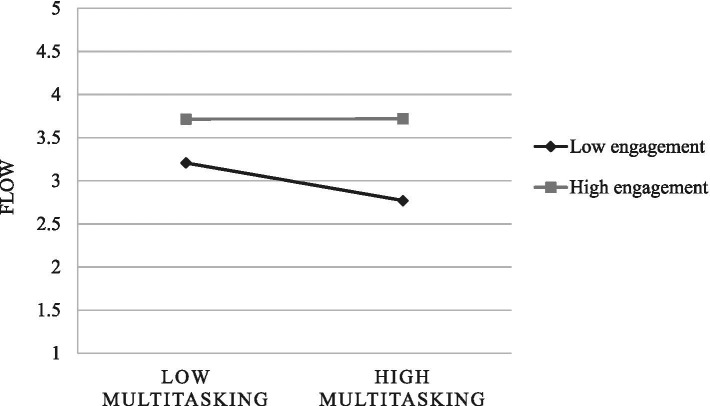
Interaction of work engagement with multitasking in predicting flow.

The next step involved testing a model that incorporates daily work engagement as a moderator of the multitasking–flow relationship. In this moderation model, both multitasking (*B* = −0.84, *p* < 0.001, *β* = −0.17) and engagement (*B* = 0.53, *p* < 0.001, *β* = 0.59) had significant main effects on flow. In addition, the interaction between multitasking and work engagement was significant in predicting flow (*B* = 1.25, *p* = 0.027). This result lends support to Hypothesis 3. The interactive effect is shown in Figure 2, further explored using the simple slopes procedure described by Preacher et al. (2006). Simple slopes were calculated for conditional values of the moderator at ±1 *SD*. Tests of simple slopes indicated that the effect of multitasking on flow was significant at lower (−1*SD*) levels of work engagement (simple slope = −1.69, *p* = 0.003) and at average levels of work engagement (simple slope = −0.84, *p* = 0.017). At higher (+1*SD*) levels of work engagement, multitasking did not significantly reduce flow (simple slope = 0.01, *p* = 0.985). We also calculated the region of significance of the simple slopes, which defines the specific values of the moderator at which the slope is statistically significant. We found that the simple slope of flow regressed on multitasking was significant for any value of work engagement below 0.11, and centered scores ranged from −1.79 to 2.00. In other words, moderate to high levels of daily work engagement buffered the detrimental effect of multitasking on flow.

Finally, we regressed performance on flow. In support of Hypothesis 4, we found that on days when employees experienced more flow, they reported higher performance compared with days on which they experienced less flow (*B* = 0.65, *p* < 0.001, *β* = 0.46). We again used the procedures of Bauer et al. (2006), this time to test a model in which flow mediates the association between multitasking and daily performance (Model 3 in Table 3). We observed that multitasking was negatively associated with flow (*B* = −1.19, *p* = 0.001, *β* = −0.25) and flow was positively associated with daily performance (*B* = 0.69, *p* < 0.001, *β* = 0.49). Thus, on days when employees multitasked more, they experienced less flow and in turn reported lower performance, compared with days on which they multitasked less. This indirect effect was estimated at −0.82 with a 95% CI [−1.38, −0.34]. Together with the mediating mechanism of challenge appraisals of daily tasks that we found earlier, this result supports Hypothesis 5.

## 5 Discussion

Today, many – if not most – employees are forced to shift attention between a high number of tasks on a daily basis, perhaps even get addicted to multitasking (Dean and Webb, 2011), yet at what cost? This paper aimed to investigate the implications of multitasking for individuals and organizations. We have argued that a better understanding of the consequences of multitasking requires (a) an ecologically valid examination of the way in which daily tasks unfold, (b) an integration of scholarly work on multitasking and work-related flow, and (c) identification of mediators and moderators that contextualize employees’ responses to multitasking. Hence, the present diary study developed and tested a comprehensive model of day-level multitasking that aimed to shed light on *why* and *when* performance of multiple tasks relates to flow and, consequently, to subjective performance at work. Our results provide support for most of the hypotheses advanced herein.

The results of our study have important implications for research on the consequences of multitasking. In a sample of professional workers, we observed that on days when employees’ working time was highly fragmented across a high number of tasks, they experienced less flow and, in turn, their self-reported performance was lower for that particular day. Adler and Benbunan-Fich (2013) posited that people who are in flow are totally focused on a single task and unlikely to multitask. Our results speak to this notion as they indicate that day-level multitasking indeed impairs flow.

We further contribute to Peifer and Zipp’s (2019) line of research and theoretical model (i.e., transactional model of stress and flow) in three important ways. First, we have put forward (and empirically tested) a mediating mechanism that underlies the relationship between multitasking and flow, focusing on appraisal of daily tasks as challenging or hindering. We found that an explanation for this relationship is that a working day full of multitasking impairs the perception of one’s daily tasks as challenges or opportunities. On days when employees’ time for tasks was highly fragmented, those tasks were appraised as less challenging, which resulted in less flow experiences. This result is in line with previous research on how flow experiences are particularly observed in challenging tasks (see Peifer and Zipp, 2019). For instance, Csikszentmihalyi (2000) described how surgeons find flow during difficult surgeries. We add to previous research the insight that multitasking as a stressor makes it more difficult to appraise one’s tasks as challenging tasks. In other words, on days characterized by multitasking, employees are less inclined to appraise their daily tasks as challenging.

Importantly, we found no such effect for the hindrance appraisal of daily tasks. While interruptions as such are typically appraised as hindrances (Smith et al., 2022), this does not necessarily mean that the interrupted and interrupting tasks are considered hindrances. Our result suggests that the hindrance of performing multiple tasks does not add to the pressure associated with a particular task or turn these tasks into hassles. Rather, multitasking is detrimental only in the sense that it interferes with the potential for achievement and personal growth of certain daily tasks (i.e., challenging tasks are not perceived as such). Hindrance appraisal of daily tasks was not related to flow, which is in line with the meta-analytic result of Liu et al. (2023) that hindrance demands were not significantly related to flow or any of its subdimensions. Conceptually, flow is more closely linked to challenges (Peifer and Zipp, 2019), and it indeed seems that only challenge appraisals can link multitasking to flow.

A second way in which we build on Peifer and Zipp’s (2019) study is that we have illuminated the ‘when’ of the multitasking–flow relationship by empirically testing daily engagement as a buffer for this relationship. It is not impossible to experience flow while multitasking. Our data indicated that employees are not equally engaged at work across days; there are days when employees feel more energetic and dedicated than on other days. Importantly, Sonnentag et al. (2010) noted that “in many work settings there are specific times and periods when it is necessary that employees are particularly engaged at work” (p. 27). Work engagement may buffer flow-inhibitors and may build resources that could activate flow-enablers (Yan and Donaldson, 2023). Our results speak to this notion, showing that on days when employees were highly engaged, the detrimental impact of multitasking on flow was weakened. It appears that daily engagement offers employees substitute resources to cope with day-level multitasking and remain in a flow state despite the demands of multitasking. Given that work engagement is a positive and high-activation state (Bakker et al., 2011), in a multitasking environment, engaged employees should be more willing to put in the effort (e.g., to navigate attention back to a task) and have abundant energy to do so (e.g., in fact navigating back to a task), much like conscientious individuals are likely to do (Demerouti, 2006). Thus, even though engaged employees have to multitask as much as their less engaged counterparts, they have the capacity and willpower to create conditions under which flow can be attained and maintained.

Third, we have addressed Peifer and Zipp’s (2019) call for studying objective multitasking demands. We consider it a key strength of our study that we relied on a fine-grained measure of multitasking that is based on daily diaries. We have investigated this phenomenon in an organizational setting, focusing on the daily working life of employees and examining how they feel they are performing in light of the multitasking reality of their organization. Thus, from a methodological point of view, we believe our results offer ecologically valid insights into why and when day-level multitasking hinders flow and subjective performance on a given day.

Our study contributes to the broad field of multitasking, as we believe the theorizing and empirical results presented in this paper may be useful in extending frameworks for the study of cumulative interruptions and task switches (see Baethge et al., 2015; Puranik et al., 2020). By first demonstrating that impaired flow explains the effect of multitasking on daily job performance, and then illuminating why (i.e., reduced challenge appraisals) and when (i.e., on days when employees are less engaged) day-level multitasking is detrimental to flow, we contribute to a more comprehensive understanding of employees’ short-term responses to juggling multiple tasks. The ‘why’ and ‘when’ of the multitasking–flow relationship relate to the primary and secondary appraisal processes in the transactional model of stress and flow (Peifer and Zipp, 2019). With regard to primary appraisal, we found that performing multiple tasks reduces the challenges one perceives throughout the workday, thereby hindering flow because flow tends to be experienced in challenging tasks. In accordance with the challenge-hindrance stressor framework (LePine et al., 2005), we argue that multitasking can be categorized as a hindrance stressor, for which employees need to adopt a coping strategy if they want to experience flow. Our study suggests that daily work engagement may provide the necessary coping resources, allowing for a more positive secondary appraisal. We believe these are insights that help further specify the transactional model of stress and flow.

Finally, the present study advances our understanding of the antecedents and consequences of flow. We have shown that flow, as an experiential well-being state (Ilies et al., 2017), has immediate implications for how employees perform. While research has established the importance of flow for predicting performance, our study adds to a small but growing body of research that examines flow as a within-person performance process (Peifer and Zipp, 2019; Soriano et al., 2021; Weintraub et al., 2021; Gerpott et al., 2022; Aitken et al., 2023). Importantly, we have identified multitasking as a major obstacle to work-related flow, at least for those employees who do not feel particularly engaged. Our study thus provides empirical support for the notion that entering flow requires initial energy (which might get lost when multitasking), but once reached it is a state in which people can recover and build resources (see also Gerpott et al., 2022).

### 5.1 Practical implications

The results from this study suggest that multitasking poses serious concerns for employee effectiveness. Notably, we observed almost no between-individual variance in day-level multitasking (see Table 1). Thus, the level of multitasking cannot be explained by any individual-level differences (such as personality or work style) but rather is determined by situational, day-level variables. In other words, multitasking is not a given or stable work feature; some days are characterized by more alternations between activities and will require more (simultaneous or sequential) multitasking behaviors than other days. In a way, this suggests that employees and managers are left with little opportunity to be proactive and optimize the daily work environment. However, Bakker and Van Woerkom (2017) claimed that flow should not be seen as a passively determined state but can be shaped by individual behaviors (see also Liu et al., 2023). In a similar vein, we believe that the level of multitasking should not be seen as something employees and managers have no control over. Employees need to realize they are active agents; they can develop strategies for managing cumulative alternations and opt for a flow-conducive strategy to cope with multiple tasks (which multitasking is not; Peifer and Zipp, 2019). In fact, optimizing job demands (i.e., simplifying the job and bypassing inefficient work processes) may be more fruitful than minimizing job demands (Demerouti and Peeters, 2018). For managers, it is important to realize that if employees are spread too thin, this limits the learning potential of challenging tasks. They should therefore be mindful of daily variations in multitasking and assist employees on those days when they have to juggle many tasks.

Such assistance could be reflected in promoting strategies for time and attention management. Specifically, organizations are recommended to enable employees to monotask more frequently and for longer stretches of time, for instance by creating spaces designated for focused, uninterrupted work. Moreover, anecdotal evidence from our study suggests that organizations should try to limit the number of meetings, as these prompted many of the task switches. Evitable work switches may be reduced also by implementing new ways of working that enable employees to work whenever and wherever they want, in particular when they need time to work on solitary projects, thereby distancing themselves physically from colleagues and clients. Such organizational interventions could enhance the restorativeness of the work environment (see Bellini et al., 2023).

Working from home may help in shaping more engaged subsequent workdays (Darouei and Pluut, 2021), which will assist employees in coping with multitasking, as our results suggest. That being said, teleworkers may struggle with homeplace intrusions and social isolation as impediments to flow (Aitken et al., 2023). Personal resources (such as discipline and resilience) seem critical in this respect. Therefore, we recommend that organizations offer personal resources interventions and mindfulness trainings for employees who are regularly faced with the demands of multitasking. An intervention study found that personal resources had a positive impact on work engagement (Bakker and van Wingerden, 2021), while mindfulness has been found to result in improved self-regulation (Glomb et al., 2011) and work engagement (Leroy et al., 2013). Mindfulness seems to be fundamentally connected to workplace functioning, especially in light of the multitasking reality of many organizations. The growing body of research on mindfulness interventions suggests that working mindfully is associated with attentional stability (sustaining attention), attentional control (directing attention amid competing demands), and attentional efficiency (economical use of attentional resources) (Good et al., 2016). When the workday involves multiple tasks, those benefits of working mindfully should assist employees in alternating effectively between activities, finding focus in their work, and determining which tasks should be completed with undivided attention.

### 5.2 Limitations and directions for future research

We should note several limitations of our study. Although multitasking was measured based on detailed reports of work activities (*cf*. perceptual measures; e.g., Kirchberg et al., 2015; Peifer and Zipp, 2019), we did not use in-the-moment data collection. We did not have information on the share of resources other than time allocated to activities, how often employees were interrupted, if and when they resumed their tasks, or the reasons for multitasking. Thus, we are somewhat limited in our understanding of the nature of employees’ multitasking behaviors and it remains unclear whether flow is hindered by the performance of multiple tasks simultaneously, the interruptions associated with multitasking, or the task-switching aspects. We therefore recommend that future studies distinguish between the switching, interleaving, and overlaying types of multitasking behavior (Circella et al., 2012). Higher levels of granularity would allow for examining whether challenge and hindrance appraisals differ across types and what are the consequences for flow and job performance. Future studies are also recommended to use observation methods (see Wajcman and Rose, 2011) or a fixed format in the survey by which employees log the start time, end time, and resumption time of all daily tasks (see Feldman and Greenway, 2021), if feasible.

Despite this limitation, our study design and data collection method were still intensive and demanding, which has come at the cost of the number of participants in the sample and the number of daily records in the analysis. Given the small sample size, it is important to interpret the magnitude of the effect sizes reported herein with caution. Also, inter-item correlations can be unstable if the sample size is small (Kennedy, 2022) and the internal consistency of a scale can decrease if items are dropped (Schaufeli et al., 2019). We indeed observed in our study that day-specific Cronbach’s alphas are quite sensitive to small sample sizes and shortened scales. Hence, we spur researchers to investigate the relationships proposed herein and replicate our results using a large sample, ideally with multiple measurements throughout the day. That is, our results may be subject to concerns regarding the possibility of reversed causation and common method bias (Siemsen et al., 2010), as our design did not involve temporal separation of the daily measures and we relied merely on employees’ self-reports for the assessment of job performance.

Our daily diary design covered 4 weeks and we were therefore not able to take a long-term perspective on the consequences of multitasking. Notably, multitasking is considered a poor strategy for learning (Rosen, 2008), and the results of the current study on challenge appraisal speak to this notion. Our suggestion is that researchers adopt longitudinal designs and examine the implications of day-level multitasking and flow for longer-term outcomes such as workplace learning. Moreover, we have exclusively focused on outcomes in the work domain. It would be interesting for future researchers to examine multitasking as a hindrance demand that depletes (personal) resources, thereby spilling over to the home domain and potentially leading to work–family conflict. We further believe that research in the field of job crafting can make a contribution to the literature on multitasking by identifying crafting behaviors of individuals (mostly likely in the domains of task and relationship crafting, see Wrzesniewski and Dutton, 2001) that serve as responses to organizational realities of multitasking. Finally, while we focused on state work engagement, trait work engagement may also prove promising to study as a person-level resource that buffers the detrimental impact of multitasking (see Puranik et al., 2020) as well as its relationship with job crafting in this context (see Bakker et al., 2023).

## Data availability statement

The raw data supporting the conclusions of this article will be made available by the authors, without undue reservation upon request.

## Ethics statement

Ethical review and approval was not required for the study on human participants in accordance with the local legislation and institutional requirements. Written informed consent from the patients/participants or patients/participants legal guardian/next of kin was not required to participate in this study in accordance with the national legislation and the institutional requirements.

## Author contributions

HP: Conceptualization, Formal analysis, Investigation, Methodology, Writing – original draft, Writing – review & editing. MD: Conceptualization, Investigation, Methodology, Writing – original draft, Writing – review & editing. MZ: Conceptualization, Investigation, Methodology, Writing – original draft, Writing – review & editing.
